# Wnt/β-catenin signaling is required for development of the exocrine pancreas

**DOI:** 10.1186/1471-213X-7-4

**Published:** 2007-01-12

**Authors:** James M Wells, Farzad Esni, Gregory P Boivin, Bruce J Aronow, William Stuart, Chelsea Combs, Angela Sklenka, Steven D Leach, Andrew M Lowy

**Affiliations:** 1 Department of Developmental Biology, Cincinnati Children's Hospital Research 45267, Cincinnati, OH 45267, USA; 2 The Department of Surgery, The Johns Hopkins University School of Medicine, Baltimore, MD 21210, USA; 3 Department of Pathology and Laboratory Medicine, USA; 4 Department of Surgery, Division of Surgical Oncology, University of Cincinnati College of Medicine, Cincinnati, OH 45267, USA

## Abstract

**Background:**

β-catenin is an essential mediator of canonical Wnt signaling and a central component of the cadherin-catenin epithelial adhesion complex. Dysregulation of β-catenin expression has been described in pancreatic neoplasia. Newly published studies have suggested that β-catenin is critical for normal pancreatic development although these reports reached somewhat different conclusions. In addition, the molecular mechanisms by which loss of β-catenin affects pancreas development are not well understood. The goals of this study then were; 1] to further investigate the role of β-catenin in pancreatic development using a conditional knockout approach and 2] to identify possible mechanisms by which loss of β-catenin disrupts pancreatic development. A *Pdx1-cre *mouse line was used to delete a floxed *β-catenin *allele specifically in the developing pancreas, and embryonic pancreata were studied by immunohistochemistry and microarray analysis.

**Results:**

*Pdx1-cre *floxed *β-catenin *animals were viable but demonstrated small body size and shortened median survival. The pancreata from knockout mice were hypoplastic and histologically demonstrated a striking paucity of exocrine pancreas, acinar to duct metaplasia, but generally intact pancreatic islets containing all lineages of endocrine cells. In animals with extensive acinar hypoplasia, putative hepatocyte transdifferention was occasionally observed. Obvious and uniform pancreatic hypoplasia was observed by embryonic day E16.5. Transcriptional profiling of *Pdx1-cre *floxed *β-catenin *embryonic pancreata at E14.5, before there was a morphological phenotype, revealed significant decreases in the β-catenin target gene *N-myc*, and the basic HLH transcription factor *PTF1*, and an increase of several pancreatic zymogens compared to control animals. By E16.5, there was a dramatic loss of exocrine markers and an increase in *Hoxb4*, which is normally expressed anterior to the pancreas.

**Conclusion:**

We conclude that β-catenin expression is required for development of the exocrine pancreas, but is not required for development of the endocrine compartment. In contrast, β-catenin/Wnt signaling appears to be critical for proliferation of PTF1+ nascent acinar cells and may also function, in part, to maintain an undifferentiated state in exocrine/acinar cell precursors. Finally, β-catenin may be required to maintain positional identity of the pancreatic endoderm along the anterior-posterior axis. This data is consistent with the findings of frequent *β-catenin *mutations in carcinomas of acinar cell lineage seen in humans.

## Background

Over the past several years, key transcription factors and signaling pathways that mediate pancreatic development have become increasingly well-defined [1]. The canonical Wnt signaling pathway has been shown to play a critical role in the development of numerous tissues, and when inappropriately activated, it plays a central role in tumorigenesis [2-5]. Prior studies have suggested the importance of Wnt signaling in pancreatic development, as expression of *Wnt1 *under control of the *Pdx-1 *promoter was associated with murine pancreatic agenesis [6]. Another study demonstrated that numerous Wnt pathway genes are expressed during pancreatic organogenesis [7]. Recently published studies from two laboratories examined the effects of deleting β-catenin, the central mediator of canonical Wnt signaling, in the mouse pancreas and reported somewhat conflicting findings. One study suggested that β-catenin/Wnt signaling was essential for development of exocrine pancreas, but played no role in endocrine development, while the other concluded that the loss of β-catenin/Wnt signaling in the developing mouse resulted in transient pancreatitis, but ultimately found that exocrine pancreas eventually recovered [8,9]. Furthermore, this study found a decrease in islet cell numbers in β-catenin knockout mice suggesting a significant role for the Wnt pathway in endocrine lineage development. It is still not clear why these reports reached different conclusions, nor have the molecular pathways that act downstream of β-catenin in the pancreas been identified.

We have utilized similar transgenic methods to delete β-catenin expression from the developing mouse pancreas. In addition, we have examined the effects of blocking Wnt signaling in dorsal pancreatic explants using a specific biochemical inhibitor PKF118–310. Finally we comprehensively investigated the molecular consequences of deletion of β-catenin on embryonic pancreas development using transcriptional profiling. We analyzed embryonic pancreata at E14.5, before the pancreas was phenotypically affected, and at E16.5, when hypoplasia of the exocrine pancreas is evident. Our studies reveal that deletion of β-catenin during pancreatic development results in decreased body size of the knockout animals in association with severe pancreatic hypoplasia and shortened median survival. The hypoplastic pancreas is marked by striking absence of exocrine mass, with preservation of pancreatic islets. Pancreatic buds exposed to a Wnt inhibitor also demonstrate decreased expression of exocrine-specific genes, suggesting that the Wnt signaling function of β-catenin is responsible for the defect in exocrine development. Proliferation of exocrine cells is significantly reduced, suggesting that Wnt signaling promotes exocrine cell proliferation during development. Transcriptional profiling and quantitative RT-PCR of embryonic pancreata reveal significant down regulation of the transcription factor *PTF1 *as early as E12.5. Down regulation of the Wnt target gene *N-myc *and the *FGFR2 *gene was observed in knockout animals which correlates with the decreased proliferation of nascent exocrine cells and may be responsible for the observed phenotype [10]. Interestingly at E14.5, there is also a significant increase in the levels of the exocrine genes elastase, and amylase, suggesting that the absence of a Wnt signal may cause precocious differentiation of exocrine progenitor cells. Together, our findings strongly support a critical role for β-catenin in exocrine, but not endocrine development and suggest several Wnt-dependent putative regulators of exocrine cell proliferation and differentiation during normal pancreatic development.

## Methods

### Generation of genetically modified mice

Mice containing a floxed allele of *β-catenin *were a generous gift of the Birchmeier laboratory. The *β-catenin *allele in this animal contains LoxP sites in the second and sixth intron as described [11]. To obtain conditional deletion of β-catenin within the pancreas, we utilized a *Pdx1-cre *transgenic strain, developed in our laboratory, and previously reported to mediate efficient recombination in the developing mouse pancreas by E10.5 [12]. Mice were genotyped using PCR on digests of ear clippings. To identify *Cre*, the following primers were used to amplify a 475-bp product: forward 5'-AGATGTTCGCGATTATCTTC-3'; reverse 5'-AGCTACACCAGAGACGG-3'.

The PCR conditions were 94°C for 3 min, then 30 cycles at 94°C for 30 s, 58°C for 45 s, and 72°C for 45 s, followed by 72°C for 5 min. To identify β-catenin lox animals, the following primers amplify a 150-bp product for wild-type, and a 200-bp product for lox mice: forward 5'-ACTGCCTTTGTTCTCTTCCCTTCTG-3'; reverse 5'-CAGCCAAGGAGAGCAGGTGAGG-3' [11]. The PCR conditions were 94°C for 3 min, then 30 cycles at 94°C for 30 s, 63°C for 45 s, and 72°C for 45 s, followed by 72°C for 5 min. The addition of a final concentration of 1.2 M betaine [Sigma-Aldrich, St. Louis, MO] enhanced the reaction. Timed pregnancies were determined by estimating that the detection of vaginal plugs occurred on E 0.5.

### Tissue processing

Mice were euthanized using isoflurane. Pancreata were dissected and placed in PBS, fixed in formalin followed by processed in paraffin wax and sectioned at 4 μm. For frozen sections, tissues were fixed for 2 hours at 4°C in 4% paraformaldehyde, washed in cold PBS and placed overnight in 30% sucrose [w/v] in PBS, prior to embedding and freezing in OCT.

### Immunostaining

Dewaxed sections were subjected to antigen unmasking with Citrate solution pH 6. [Invitrogen Inc., Carlsbad, CA]. Slides were incubated in 0.3% hydrogen peroxide/methanol and then blocked with Beat Block, from HistoMouse max kit [Invitrogen Inc.] Primary antibody Anti-Active-β-Catenin [anti-ABC] clone 8E7 [Upstate, Charlottesville, VA] at 1:100 was applied O/N, 4°C. Secondary antibody, chromagen and counterstain were provided and the manufacturer's protocol was used. Primary antibodies to albumin [Abcam Inc., Cambridge, MA] and amylase [Sigma-Aldrich, St. Louis, MO] were incubated with slides at 1:3000 and 1:500, respectively, 1 hr R/T after citrate epitope retrieval, avidin and biotin blocking [Invitrogen Inc.]. Nonspecific binding was blocked with appropriate serum incubations for 30 minutes. Biotin conjugated secondary antibodies [Invitrogen, Inc.] were applied 30 minutes R/T followed by incubation with HRP Streptavidin 15 minutes and visualized with DAB [3, 3-diaminobenzidine, Dako, Carpinteria, CA]. They were counterstained with Mayer hematoxylin. Apoptotic cells were detected by flourescein in situ tunnel method, TACS TdT kit [R&D Systems, Minneapolis, MN]. Proteinase K was used for epitope retrieval and all steps were performed according to manufacturer's protocol.

### Immunofluorescent staining

For immunolabeling, tissues were sectioned and fixed in 4% PFA for 15 minutes at room temperature, washed in PBS and stained as previously described [Esni et al., 2001]. The following antibodies were used at the indicated dilutions; rat monoclonal anti-E-cadherin [Zymed Laboratories, 1:200], goat polyclonal anti-amylase [Santa Cruz Biotechnology, 1:500], goat polyclonal anti-β-catenin [Santa Cruz Biotechnology, 1:100], guinea pig polyclonal anti-insulin [Linco Research, 1:1000], rabbit polyclonal anti-Pdx1 [CHEMICON International, 1:250] rabbit polyclonal anti-PTF1a-p48 [gift from Dr. Helena Edlund, 1:1000], rabbit anti-phosphohistone H3 [Upstate 65–570, 1:200]. The following reagents were purchased from Jackson ImmunoResearch Laboratories: Biotin-conjugated anti-goat, anti-guinea pig [1:250, 1:500], Cy3-conjugated anti-rabbit [1:300], Cy2-conjugated anti-rat [1:300], and Cy2-, Cy3-, C5-conjugated streptavidin [1:300, 1:1000, 1:100].

### Explant culture of embryonic pancreas

Isolation and culture of pancreatic rudiments were carried out essentially as previously described [13]. Intact E10.5 dorsal pancreatic buds were dissected and cultured on Millipore^® ^filters for 7 days in culture medium [BioWhittaker Medium 199, 10% fetal calf serum, 50 U/ml penicillin G-streptomycin, 1.25 ug/ml Fungizone^®^] in the presence or absence of 20 nM Wnt-pathway inhibitor compound PKF118–310 [ref]. Culture medium was changed every day and by the end of the culture period, explants were either embedded in OCT and frozen in liquid nitrogen for immunostaining analysis or put in TRIzol^® ^Reagent for total RNA extraction.

### RNA isolation and microarray hybridization

Pancreata were dissected from 12.5, 14.5 and 16.5 day embryos taken from timed matings of *Pdx1-cre *floxed *β-catenin *mice and immediately submerged in RNA*later *[Ambion, Inc., Austin, TX]. Total RNA was purified using RNeasy columns [Qiagen, Valencia, CA] according to the manufacturer's directions and quality was assessed using an Agilent 2100 Bioanalyzer [Agilent Technologies, Inc., Palo Alto, CA]. 120 ng of total RNA from 2 wild type [WT] and 4 *β-catenin *knock-out [KO] pancreata at the 14.5 and 16.5 time points was amplified twice with the Arcturus RiboAmp kit [Arcturus Bioscience, Inc., Mountain View, CA] and cRNA was labeled with biotin-UTP, CTP using T7 RNA polymerase [Enzo Life Sciences, Inc., Farmingdale, NY]. Biotinylated cRNA from each embryonic pancreas was purified with RNeasy columns and hybridized to an Affymetrix GeneChip mouse genome 430 v2.0 array and stained using standard procedures [Affymetrix, Santa Clara, CA]. The 430 v2.0 array contains 45,000 probe sets representing over 34,000 mouse genes.

### Bioinformatics

We sought to identify genes whose expression was lost or gained as a result of loss of β-catenin gene expression. To do this, microarrays were generated from RNAs isolated from independent wildtype and knockout embryos as previously described. Affymetrix MOE430plus2 microarray Cel files generated from GCOS 5.0 were subjected to RMA normalization as implemented in GeneSpring 7.1. All E14.5 genechip RMA values were converted to the ratio relative the average of the corresponding day wildtype values. In this fashion one does not see the effect of increased or decreased expression in WT E16.5 relative to WT E14.5. Probe sets were first filtered for those that are overexpressed or underexpressed in knockout animals at E14.5 [486 probesets; initial criteria over 1.25 in 3 of 4 samples or under 0.66 in 3], and the same for E16.5 [513 probesets, initial criteria over 1.33 in 4 or under 0.5 in 4]. These two lists were combined [967] and then subjected to statistical analysis differentially expression in knockouts with p < 0.05 using the Students T-test assumption of equal distribution and Benjamini-Hochberg false discovery corrected p-values. This procedure generated a list of 770 probe sets. Log2 gene expression ratios were then subjected to hierachical clustering using the standard correlation distance metric as implemented in GeneSpring. Individual clusters were then analyzed for functional relatedness of genes within the cluster using David 2.0.

### Quantitative RT-PCR

For quantitative RT-PCR, total RNA was purified and characterized as described above. 500 to 1000 ng of RNA was converted to cDNA using random hexamers and Superscript III [Invitrogen, Carlsbad, CA]. Amplification was carried out with an ABI 7300 real time PCR system [Applied Biosystems, Foster City, CA] using SYBR green and the standard protocol. The reference gene was β-glucuronidase for all genes except Ptf1, for which Gapdh was used. Data was normalized using the delta-delta-Ct method. Whenever possible, primers were designed to span an intronic sequence and were validated by PCR and gel analysis.

Primer sequences were as follows:

β-glucuronidase

5'-TTGAGAACTGGTATAAGACGCATCAG-3' forward

5'-TCTGGTACTCCTCACTGAACATGC-3' reverse

Gapdh

5'-AATGGTGAAGGTCGGTGTG-3' forward

5' GAAGATGGTGATGGGCTTCC 3' reverse

Amylase

5'-GGTTCTCCCAAGGAAGCAG-3' forward

5'-TGTCACACGGCCATTTCC-3' reverse

Elastase

5'-CATCCAGACAGCTTGCCTC-3' forward

5'-CTCAGGGTGTCAGGACTGTTC-3' reverse

Fgfr2

5'-AAGCAGGAGCATCGCATTGG-3' forward

5'-TGACGGGACCACACTTTCCATA-3' reverse

N-myc

5'-CTCAAGTCAGTGCAGGCGAG-3' forward

5'-CCACCGTTACGACATCAATCTC-3' reverse

Ptf1

5'-CAGGCCCAGAAGGTTATCATCTG-3' forward

5'-AGGAAAGAGAGTGCCCTGCAAG-3' reverse

## Results

### β-catenin knockout results in small body size, severe pancreatic hypoplasia and early lethality

**Figure 1:**
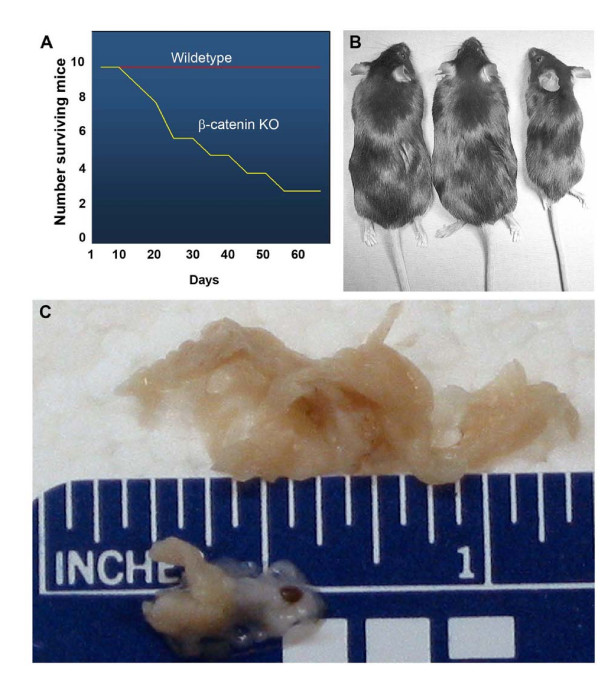
**Pancreas-specific deletion of β-catenin results in shortened lifespan and a hypoplastic gland**. β-catenin KO mice have a median survival of 29 days [A], reduced body size [B] and the pancreas averages 30% of the weight of a wildtype organ [C].

We achieved pancreas-specific deletion of β-catenin by breeding a *Pdx1-cre *strain developed in our laboratory with a strain containing a floxed *β-catenin *allele in the second and sixth intron [11,12]. *Pdx1-cre *mice heterozygous for the floxed *β-catenin *allele displayed no apparent abnormalities in either the embryonic or adult pancreas. Mice with conditional knockout of both *β-catenin *alleles in the pancreas [*Pdx1-cre *floxed *β-catenin*] were viable, but demonstrated small body size at birth that persisted into adulthood. For simplicity, these animals are referred to as β-catenin KO in the remainder of the text. β-catenin KO mice showed no obvious defects in their ability to nurse or wean, but had a significantly shortened lifespan, with a median survival of 29 days [Figure 1A–B]. Several mice did survive to greater than 6 months. Necropsy revealed no gross defects in development of the stomach, duodenum, colon, spleen or other organs. The pancreata, however, were severely hypoplastic, averaging 30% of the weight of pancreata from wildtype littermates [Figure 1C]. Although the cause of death is not known, we have determined that it is not due to defects in endocrine pancreas function [discussed below].

### β-catenin knockout results in loss of exocrine pancreatic tissue

**Figure 2:**
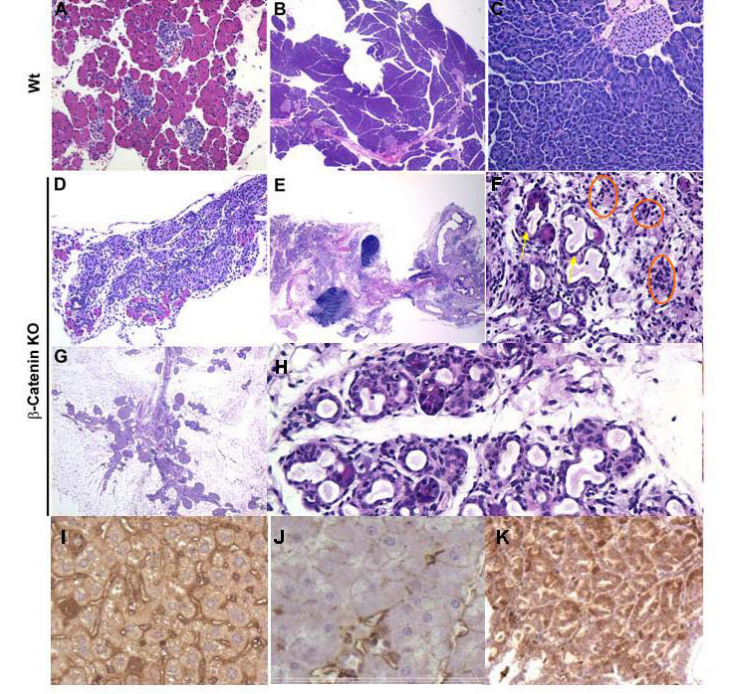
**β-catenin KO pancreata display extensive loss of exocrine tissue**. Hematoxylin and eosin stained paraffin sections were used to compare the histology of wildtype versus β-catenin KO pancreata at one day, one month and two months of age. Compared to one day old wildtype pancreas [A], the absence of acini is readily apparent at low power [D]. At two months of age, compared to wildtype [B, C] the β-catenin KO pancreas is hypoplastic with increased areas of interlobular fibrosis [E]. Under 20× magnification, increased formation of tubular duct-like structures are present suggesting acinar to duct metaplasia (arrows) and inflammatory infiltrate (circled) are evident [F and H]. By two months, low power views demonstrate extensive fibrosis and near complete absence of exocrine pancreatic tissue, likely accounting for the shortened lifespan seen in these animals [E and G]. In several mice aged beyond three months, areas of pancreatic parenchyma histologically resemble liver. Immunostaining for albumin is absent in wildtype pancreas  [J], but abundant in normal liver [I] and in β-catenin KO pancreata [K].

To determine the effects of β-catenin deletion on pancreatic morphology, we examined pancreata by standard H&E staining. At birth, pancreatic tissues from the conditional β-catenin knockout mice had were characterized by a striking paucity of acinar cell mass, numerous tubular structures suggesting acinar to duct metaplasia. In addition, we observed increased parenchymal fibrosis, and a variable degree of surrounding inflammatory infiltrate, which became more severe by one month of age [Figure 2A–F]. The phenotype progressed with age as the pancreata from mice surviving longer than 2 months often demonstrated near complete absence of acinar cell structures [Figure 2G–H]. The inflammatory infiltrate associated with the intralobular fibrosis was reminiscent of acute on chronic pancreatitis. In four animals of 23 (17%) examined that survived beyond three months, the pancreatic harvested at necropsy had a liver-like histology on H&E, suggesting the possibility of hepatic transdifferentiation. Immunostaining of these tissues was positive for albumin demonstrating that a liver-like differentiation program may be activated in these tissues [Figure 2I–K].

### Pancreatic islets cells are preserved in the β-catenin knockout

**Figure 3:**
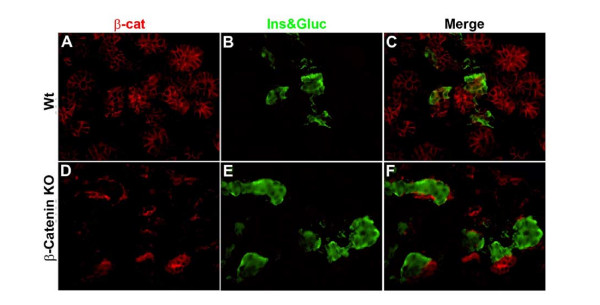
**Endocrine cells are preserved in the absence of β-catenin**. Immunofluorescent staining of E15.5 comparing wildtype [A-C] and β-catenin KO [D-F] demonstrates preservation of insulin and glucagon expressing cells in the β-catenin KO [CKO].

Pancreatic islet cell number was quantitated in wildtype and β-catenin KO mice by counting islets in serial pancreatic sections from newborn mice. We found no significant difference in islet cell numbers suggesting endocrine development is preserved in the β-catenin KO mouse [data not shown]. Endocrine cells were further characterized by immunostaining for insulin and glucagon. These studies revealed that despite the absence of β-catenin expression, insulin and glucagon cells were present and normally distributed within the islets [Fig 3A–F]. Three separate fasting glucose levels were obtained from 6 one month-old β-catenin KO mice. All mice were found to be euglycemic [data not shown]. This constellation of findings suggest that no significant defect in endocrine development following deletion of β-catenin from the developing mouse pancreas.

### β-catenin function is required prior to E16.5 in pancreatic development

**Figure 4:**
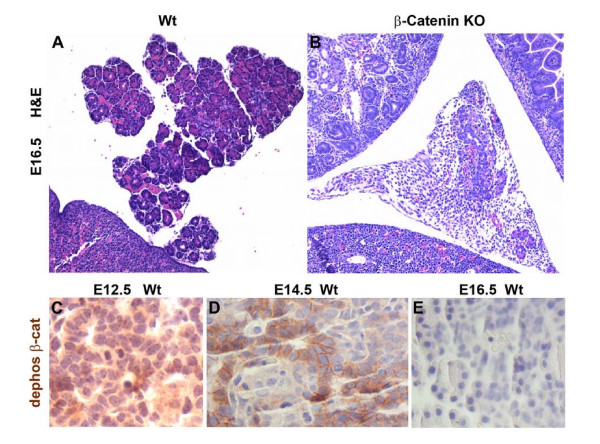
**β-catenin KO pancreas is hypoplastic by E16.5**. Panels A and B depict hematoxylin and eosin staining of E 16.5 wildtype and β-catenin KO pancreas, respectively, each at 25× magnification. Hypoplasia of the developing pancreas is already apparent and was consistently present by this stage. Panels C, D, and E demonstrate wild-type pancreata at E 12.5, 14.5 and 16.5, respectively, stained with an antibody specific for the transcriptionally active, dephosphorylated form of β-catenin. Note that staining is abundant at ED12.5, but is undetectable following the secondary transition at E16.5.

We next analyzed pancreata from β-catenin KO mice beginning at ED 12.5 to determine when pancreatic development was perturbed. We noted no obvious difference in the size of pancreata until E16.5, at which point loss of β-catenin results in an obvious and consistent decrease in pancreatic mass, corresponding to a decrease in the number of recognizable nascent acini [Figure 4A–B].

This finding led us to hypothesize that in normal pancreatic development, the presence of Wnt signaling would be critical prior to E16.5. To explore this idea, we examined embryonic pancreas for expression of the dephosphorylated, active form of β-catenin using a phospho-specific antibody. We detected activated β-catenin protein expression at E12.5 that remained at E14.5 but then rapidly disappeared by E16.5 Figure [4C–E]. Dephosphorylated β-catenin protein expression was primarily membranous and cytoplasmic but rarely nuclear, a finding also reported by Murtaugh et al. [8]. This data suggest that Wnt signaling is active in developing pancreas prior to the secondary transition and that exocrine development likely requires Wnt activity until E16.5, but not later as transcriptionally active dephosphorylated β-catenin is undetectable thereafter.

### Preserved acini express β-catenin

**Figure 5:**
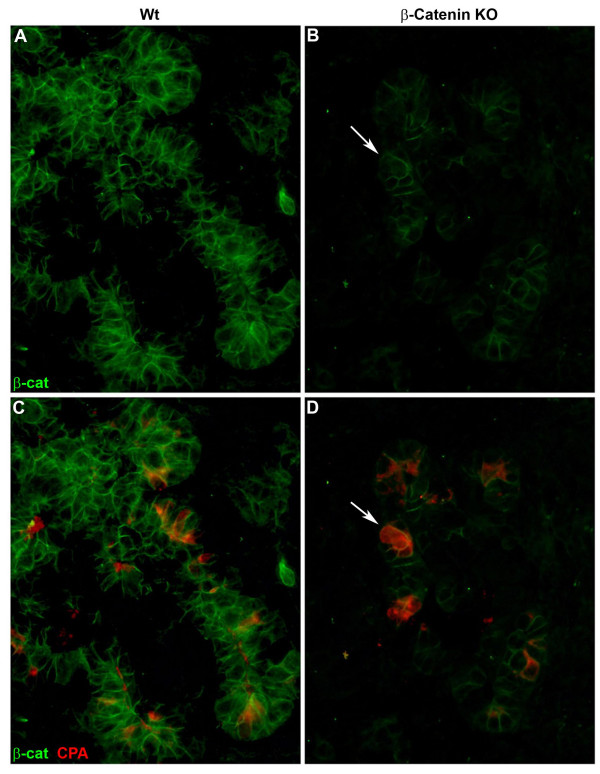
**Nascent acini in the β-catenin KO mouse expressβ-catenin**. Immunofluorescent staining of E12.5 pancreata demonstrating decreased expression of β-catenin carboxypeptidase A and amylase in the β-catenin KO [CKO] as compared to wildtype. Note that cells expressing carboxypeptidase A co-express β-catenin [arrows].

Given that the β-catenin KO was based on conditional activation of the cre recombinase expressed from a transgenic allele, it is likely that cre expression was mosaic. While the majority of the acinar pancreas was absent, it was striking that there were clusters of normal appearing acini. Therefore, we hypothesized that the presence of preserved acini would correspond to the presence of β-catenin expression. This proved to be the case as essentially all acini were found to express β-catenin. Co-staining for β-catenin/carboxypeptidase A in E 12.5 pancreata [Figure 5A] revealed that pancreatic enzyme expression is present only in cells co-expressing β-catenin. It was extremely rare (<1% of acini) to identify expression of pancreatic acini in the absence of β-catenin expression strongly suggesting the requirement o β-catenin expression for proper exocrine development.

### Pancreatic progenitor cells expressing Pdx1 are maintained in the β-catenin KO pancreas

**Figure 6:**
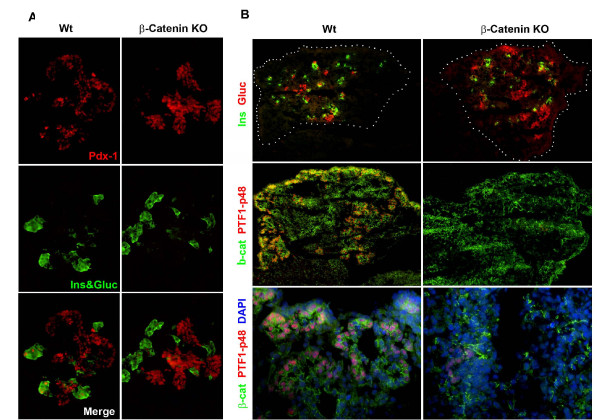
**Pdx1 expressing progenitors are preserved in β-catenin KO pancreas**. Panel A demonstrates immunofluorescent staining of E 12.5 pancreas from wildtype and β-catenin KO [CKO] revealing preservation of the Pdx1 expressing cell population. This suggests that the loss of exocrine pancreas seen later is not due to a loss of Pdx1 expressing progenitors. Panel B depicts E14.5 pancreas from wildtype and β-catenin KO stained for PTF1p48. Note the significant reduction in PTF1 expressing cells is already obvious at this developmental stage.

We next sought to evaluate whether deletion of β-catenin influenced the development of pancreatic progenitors expressing Pdx1. In other genetically modified mice, pancreatic hypoplasia has been associated with a reduction in the Pdx1-expressing progenitor cells [14]. We analyzed pancreata at E12.5 and found no difference in the number of cells expressing Pdx1 in the β-catenin KO versus the wildtype [Figure 6A]. Next, we evaluated the expression of PTF1A-p48, whose expression become restricted to exocrine cells at approximately E12.5. As early as E14.5, β-catenin KO pancreata displayed reduced expression of PTF1 protein [Figure 6B]. This data demonstrates that the defect in exocrine pancreatic development observed in the β-catenin KO is not a result of depletion of Pdx1 expressing progenitors. Instead, a population of cells marked by high PTF1A-p48 expression is severely reduced in the β-catenin KO mouse.

### Proliferation rate, not apoptosis distinguishes β-catenin KO pancreata from wildtype

**Figure 7:**
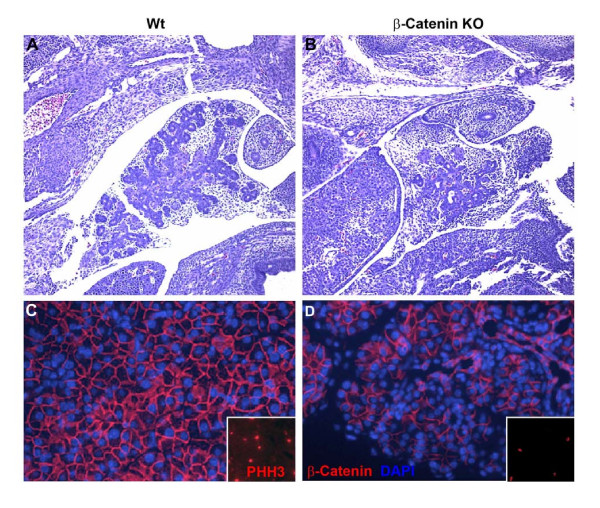
**Cell proliferation is decreased in β-catenin KO pancreas**. Panel A and B depict hematoxylin and eosin staining [25× magnification] of E 14.5 pancreata from wildtype and β-catenin KO mice. Panels C and D demonstrate costaining for β-catenin and DAPI. Insets reveal characteristic fields immunostained for phosphohistone H3, demonstrating significantly fewer proliferating cells in the β-catenin KO pancreas.

In order to determine if the loss of acinar cells in the β-catenin knockout was related to a decrease in proliferation of β-catenin-deficient cells, we performed immunostaining of β-catenin KO and wildtype pancreata at ED 14.5 using an antiphosphohistone H3 antibody [Figure 7]. These studies revealed a significantly decreased proliferation rate in the β-catenin KO versus wildtype [28.2% vs. 48.6% p < 0.05, t-test]. We also evaluated for the presence of apoptosis within the β-catenin KO animals using a TUNEL assay, but did not find any significant differences between wildtype and knockout animals [data not shown]. These findings suggest that β-catenin expression is required for proliferation, rather than apoptotic resistance of exocrine progenitors.

### Wnt inhibitors result in decreased amylase expression in dorsal pancreatic explants

**Figure 8:**
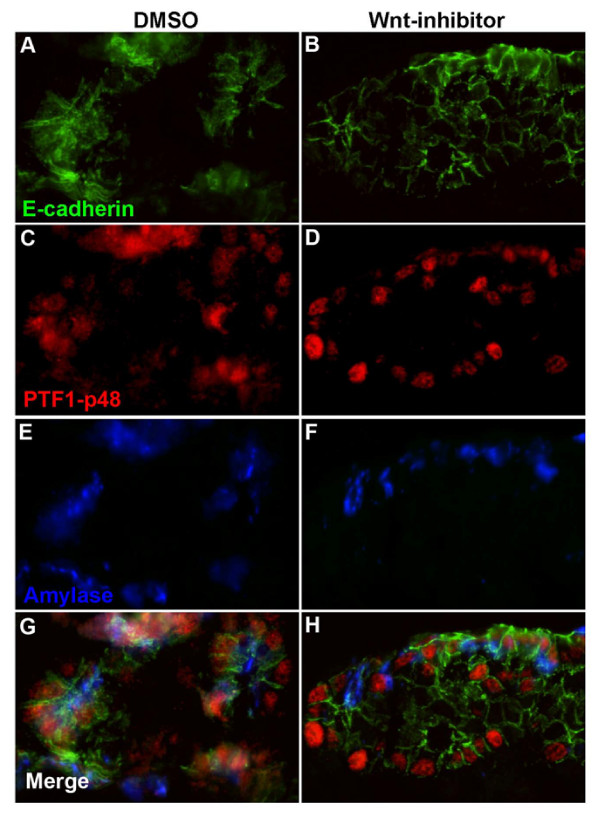
**Wnt signaling regulates exocrine-specific gene expression in cultured pancreatic explants**. Immunostaining of e10.5 dorsal pancreatic explants exposed to the Wnt specific inhibitor, PKF 118–310 [20 nM]. Explants were established and then exposed to PKF 118–310 for 7 days. Treatment resulted in decreased expression of amylase, even among PTF1-expressing cells.

Thus far, we have demonstrated that deletion of β-catenin is associated with a defect in exocrine development, possibly related to decreased proliferation of exocrine progenitors. It is probable that such a defect relates to a decrease in the Wnt signaling function of β-catenin, given the temporal expression of dephosphorylated β-catenin at this stage in the developing pancreas. However, β-catenin also functions in the E-cadherin-catenin cell adhesion complex and it is therefore possible that part of the phenotype we have observed is related to this function of β-catenin [15]. To investigate this possibility, we cultured explants from E10.5 dorsal pancreatic buds for 7 days in the presence of a specific inhibitor of β-catenin/Tcf transcription, PKF118–310 [16]. Typically, E10.5 dorsal pancreatic explant cultures robustly express exocrine markers after 7 days in culture [13]. In contrast, inhibition of Wnt signaling in dorsal bud explants results in a significant decrease in expression of amylase, similar to the findings observed in vivo [Figure 8]. When the inhibitor was applied after 3 days, no significant effect was observed, suggesting that the importance of Wnt signaling in this system is restricted to the first 3 days in culture. These data provide additional evidence that the Wnt signaling function of β-catenin is required to regulate exocrine cell proliferation and differentiation in the fetal pancreas. The persistence of PTF1 protein expression in vitro may reflect onset of its expression prior to inhibitor addition. Alternatively, loss of PTF1 expression that we observed in vivo may be regulated by a Wnt independent function of β-catenin. Finally, these experiments do not rule out a role for β-catenin in cell adhesion in vivo at later stages of development or in the adult.

### Transcriptional profiling reveals significant changes in gene expression following deletion of β-catenin

**Figure 9:**
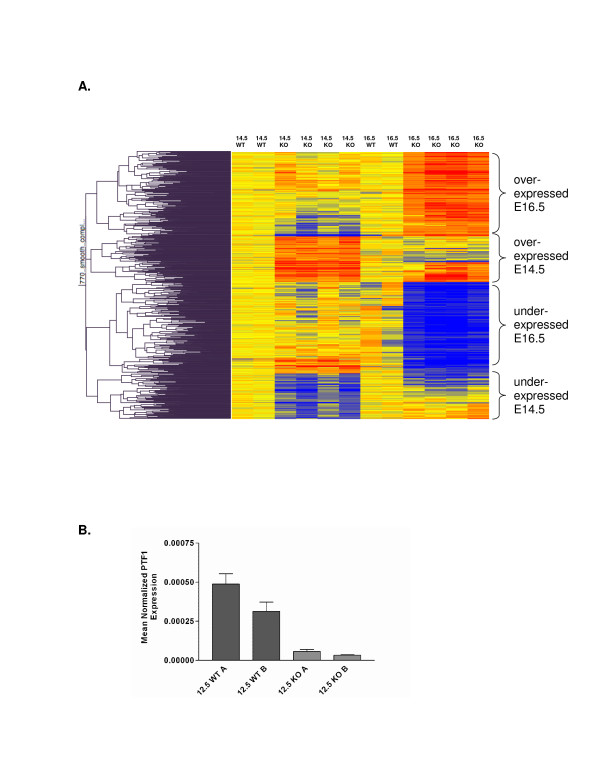
**β-catenin KO results in a characteristic transcriptional signature in E14.5 and 16.5 pancreata**. Panel A depicts a heat map demonstrating differing transcriptional profiles of Wt and β-catenin KO pancreata at E 14.5 and 16.5. Two wildtype and 4 knockout samples for each timepoint are depicted. Panel B shows quantitative RT-PCR results confirming the loss of PTF1 expression following β-catenin KO detectable as early as E12.5.

**Figure 10:**
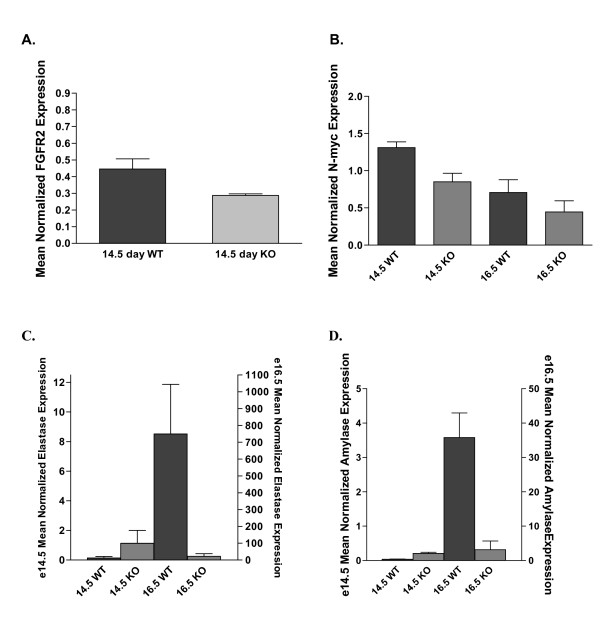
**QPCR Confirms Differential Expression of Genes Following β-catenin KO**. Panel A-D depicts QPCR assay results for FgfR2, N-myc, elastase and amylase. Transcripts for FgfR2 and N-myc are reduced in the β-catenin KO animal at E14.5. In contrast, the pancreatic enzymes elastase and amylase show increased expression at E14.5 which is reversed by E16.5.

Although β-catenin has been shown to be necessary for proper pancreas development, the molecular pathways that are perturbed downstream of β-catenin are largely unidentified. Targets of β-catenin/Tcf transcription have been demonstrated to be tissue specific [17,18]. Given our findings that β-catenin KO inhibits exocrine pancreatic development, we sought to identify targets of β-catenin/Tcf transcription that are changed in association with the β-catenin KO. We chose to assay the whole pancreas transcriptome at two timepoints, E14.5 and E16.5. These stages were chosen for two reasons, first there is a marked change in β-catenin expression around the secondary transition; expression of dephospho-β-catenin is high at E14.5 and it is absent at E16.5. Second, there is no morphological phenotype at E14.5 whereas at E16.5 there is significant exocrine hypopalsia. We harvested the embryonic pancreas from two wildtype, and four β-catenin KO mice at E14.5 and E16.5, and extracted total RNA for studies using Affymetrix GeneChips and quantitative RT-PCR [QPCR]. Analysis of the transcriptional profiles demonstrated significance differences between wildtype and β-catenin KO pancreata at each timepoint [Figure 9A, Table 1]. There was more variability in the E14.5 sample, possibly due variability in harvesting these smaller pancreata or due to stage variation as the developmental range of an E14.5 embryo is significant. Also, this is when the secondary transition is occurring and one might normally expect to see many gene expression changes at this stage of pancreatic development. As a result, we used less stringent initial criteria for filtering transcripts that might be regulated by β-catenin [see Methods]. Clustering revealed very good consistency, and in all, 770 probe sets that mapped to 645 non-redundant genes were identified as differentially expressed between wildtype and either or both of these time points. The most differentially expressed genes at each time point are summarized in Tables 1, 2, 3, 4. GeneChip and QPCR were congruent with our immunostaining findings as *Pdx1 *transcript levels did not differ between wildtype and β-catenin KO. Expression of *PTF1 *transcript was reduced in the β-catenin KO at E14.5 by microarray and as early as E12.5 by QRT-PCR [Figure 9B]. We observed no change in expression of the canonical Wnt target genes *Cyclin D1 *or *C-myc *by either RNA assay (data not shown). Instead, our studies revealed a significant decrease in expression of *N-myc *at both time points [decreased by 2 fold at E14.5]. Consistent with this, *N-myc *was only recently shown to be a direct target of Wnt signaling in the lung [10]. GeneChip studies also revealed a 1.7 fold decrease in the expression of transcripts encoding *Fgfr2 *at E14.5, consistent with the role of this receptor in exocrine pancreas development [19]. QPCR confirmed decreases in these transcripts in the β-catenin KO mouse [Figure 10A-D].

**Table 1:** Most Downregulated Genes at E14.5 Following Deletion of β-catenin in Mouse Pancreas.

**Gene**	**Fold Change**
3-phosphoglycerate dehydrogenase	9
Leucine zipper transcription factor-like 1	5.9
Adaptor-related protein complex AP-1, sigma 3	5.3
Procollagen, type IX, alpha 1	2.9
Niban protein	2.9
Hydroxyprostaglandin dehydrogenase 15 (NAD)	2.1
One cut domain, family member 1	2
Musashi homolog 2 (Drosophila)	2
Flavin containing monooxygenase 2	2
Thymopoietin	2
Solute carrier family 19 (thiamine transporter), member 2	2
Neuroblastoma myc-related oncogene 1	2
Regucalcin	2
Melanoma inhibitory activity 1	2

**Table 2:** Most Upregulated Genes at E14.5 Following Deletion of β-catenin in Mouse Pancreas.

**Gene**	**Fold Change**
Elastase 3B, pancreatic	11.3
Myosin, heavy polypeptide 4, skeletal muscle	8.8
Carboxypeptidase A3, mast cell	6
CDNA sequence BC003331	5.8
Neuropeptide Y	5.8
Hypothetical gene supported by AK087915, similar to predicted human Leucine Rich Repeat protein gene ENSG00000178125	5
Histidine decarboxylase	4.8
Insulin I	4.7
Carboxypeptidase B1 (tissue) (Cpb1), mRNA.	4.6
Elastase 3B, pancreatic	4.6
Glucose-6-phosphatase, catalytic, 2	4.2
Islet amyloid polypeptide	4.2

**Table 3:** Most Downregulated Genes at E16.5 Following Deletion of β-catenin in Mouse Pancreas.

**Gene**	**Fold Change**
RIKEN cDNA 1810010M01 gene	100
Kallikrein 6	25
Pancreatic lipase-related protein 2	20
CUB and zona pellucida-like domains 1	0.07
Cullin 4A	0.07
Ribonuclease, RNase A family, 1 (pancreatic)	12.5
Trypsin 4	11
Chymotrypsin-like	10
Elastase 3B, pancreatic	8.5
Cytochrome P450, family 2, subfamily c, polypeptide 70	7.7
Aquaporin 8	7.7
Elastase 2	7.7
Glycine N-methyltransferase	7.7
Protease, serine, 3	7.1
Regenerating islet-derived 1	6.7
Annexin A13	6.7
Cystathionine beta-synthase	6.3
Gamma-glutamyltransferase 1	6.3
Alcohol dehydrogenase, iron containing, 1	5.9
Insulin induced gene 1	5.9

**Table 4:** Most Upregulated Genes at E16.5 Following Deletion of β-catenin in Mouse Pancreas.

**Gene**	**Fold Change**
Paired-like homeobox 2b	13.4
MIC2 (monoclonal Imperial Cancer Research Fund 2)-like 1	6.1
Regulator of G-protein signaling 2	4.9
Fibulin 5	4.7
Interleukin 2 receptor, gamma chain	3.8
X-linked lymphocyte-regulated 4	3.5
Sulfatase 2	3.4
Hemoglobin Z, beta-like embryonic chain	3.4
Profilin 2	3.3
UDP-GlcNAc:betaGal beta-1,3-N-acetylglucosaminyltransferase 5	3.2
Kelch-like 4 (Drosophila)	3.2
Heart and neural crest derivatives expressed transcript 2	3.2
Secreted frizzled-related sequence protein 1	2.9

Other notable genes significantly reduced at E14.5 included musashi homolog 2 (*Msi2h*) [2 fold], an RNA binding protein implicated in maintaining the identity of progenitor cell fates in the brain [20] and hedgehog-interacting protein [*Hhip*] [1.9 fold] suggesting a potential role for this signaling pathways in exocrine pancreatic development. A transcription factor of unknown function, the leucine zipper-like transcription factor1 (*Lzltf1*), was among the most reduced genes at E14.5, with its expression being decreased by an average of 5.9 fold. *Hox b4 *was significantly increased in E16.5 β-catenin mutant pancreata suggesting that loss of Wnt signaling results in a partial homeotic transformation [21]. This is further supported by the appearance of albumin-expressing cells in the adult pancreas [Figure 2I].

Among the transcripts most down regulated in E16.5 β-catenin KO mutants included acinar cell specific genes such as pancreatic elastase (*Ela3b*), and trypsin (*Try4*). Interestingly, at E14.5, the transcript for pancreatic elastase (*Ela3b*) was upregulated an average of 11.3 fold, and the transcript for pancreatic amylase2 (*Amy2*) was upregulated an average of 2.3 fold. QPCR confirmed the upregulation of transcripts at E14.5 as compared to wildtype, and demonstrated the reversal of this pattern by E16.5 [Figure 10B]. Interestingly, immunostaining did not reveal increases in elastase or amylase protein levels at E14.5. Uniformly, however, all exocrine gene transcripts were dramatically down regulated by E16.5 compared to wildtype. These data suggest that the tight control between proliferation and differentiation is perturbed in β-catenin mutants. Despite the lack of an endocrine phenotype in the β-catenin KO mice, we did observe upregulation of transcripts for insulin I (*Ins1*) [4.7 fold], and a slight increase in glucagon (*Gcg*) [1.4 fold] at ED14.5. By 16.5, however, none of these genes were differentially expressed compared to wildtype mice. "Raw and normalized microarray data from this study can be obtained from the Gene Expression Omnibus (GEO) at the National Center for Biotechnology Information (NCBI) database [22].

## Discussion

β-catenin is the central mediator of the canonical Wnt signaling pathway and regulates many cell fate decisions during normal development in numerous tissues. Deregulation of β-catenin/Wnt signaling is a sentinel event in colorectal neoplasia and contributes to tumorigenesis in many other sites. Mutations in β-catenin have been demonstrated in exocrine pancreatic cancers, pancreaticoblastoma and solid-pseudopapillary tumors [23-25]. Our laboratory has been interested in the role of Wnt signaling in pancreatic neoplasia. Recent studies have demonstrated the reactivation of pathways critical to normal pancreatic development, such as Notch and Hedgehog, during pancreatic duct carcinogenesis thus further linking developmental pathways with pancreatic neoplasia [26-28].

Given the apparent role of β-catenin/Wnt signaling in certain pancreatic neoplasms, we hypothesized that this pathway may be an important mediator of pancreatic development. Recently, two separate reports were published that examined the effects of genetically deleting β-catenin in the developing pancreas, using similar methods to those presented in the current study. In addition, a newly published study by Heiser et al. examined the effects of stabilized β-catenin on pancreatic development [29]. While these studies utilized similar methods, their conclusions had several significant differences. The current study utilizes mouse genetics and in vitro studies of dorsal pancreatic explants, along with gene profiling studies to suggest molecular mechanisms by which β-catenin deletion effects mouse embryonic pancreas development. In summary, our studies show that β-catenin expression is required for development of the exocrine pancreas, but is not required for development of the endocrine pancreas. Our data suggests that it is inhibition of canonical Wnt signaling that effects early exocrine cell development. Lastly, our transcriptional profiling studies suggest several potential roles for β-catenin in the developing pancreas that include maintenance of undifferentiated proliferating exocrine progenitor cells and maintenance of positional identity along the anterior-posterior axis.

Our findings are highly concordant with that of Murtaugh et al. who also noted severe loss of exocrine, but preservation of endocrine pancreas following conditional knockout of β-catenin. The findings of these two studies are in contradistinction to the study by Dessimoz et al. in which only modest reversible changes in exocrine pancreatic development were present, while endocrine pancreatic development was impaired. We believe that difference in timing and levels of cre expression between different *Pdx1-cre *lines is largely responsible for the difference in phenotypes observed in our study versus the study of Dessimoz et al. The *Pdx1-cre *strain used in this study has been shown to direct cre-mediated recombination as early as ED10.5 and we often observed a near complete loss of exocrine pancreatic tissue and early lethality in β-catenin KO animals. In contrast, the Dessimoz study found that animals developed pancreatitis that recovered in adulthood. Recently, Heiser et al. found that two different *Pdx1-cre *strains caused significantly divergent phenotypes when bred to transgenic mice harboring an allele of *β-catenin *that can be stabilized in a cre-dependent fashion. The investigators noted an early and late pattern of cre expression that was associated with pancreatic hypo- versus hyperplasia, respectively. The "late" *Pdx1-cre *strain utilized in the Heiser study was the same strain used by Dessimoz et al. It follows then that this line would cause less severe perturbations in exocrine pancreatic development as observed by these investigators. Also, inactivation of β-catenin at later stages of development might uncover a role for β-catenin in endocrine cell development and explain why there were fewer endocrine cells in adult islets in the Dessimoz et al study. It is possible that β-catenin has several different roles throughout development of the pancreas and that the timing of cre-mediated activation is essential to uncover these different functions. Our findings support those of both Dessimoz and Murtaugh et al. that β-catenin, through its role in Wnt signaling, plays a role in proliferation of developing acini, rather than in apoptotic resistance.

Not surprisingly, the published and current studies confirm the previously described role for β-catenin in regulating cell proliferation. However, it is likely that there are additional downstream consequences of β-catenin deletion that remain to be identified. We have used microarrays to globally investigate the role of β-catenin in the developing pancreas. To focus on direct versus indirect effects, we analyzed embryonic pancreata lacking β-catenin before and after exocrine loss were morphologically evident. Consistent with its role in cell proliferation we found reduced levels of genes and pathways known to regulate pancreatic cell proliferation including *Ptf1*, *Fgfr2 *and *Egf *[19,30,31]. Fgfr2 is expressed in the pancreatic epithelium and is activated by Fgf10 from the mesenchyme, a signal that is important for proliferating pancreatic progenitor cells [19,32,33]. Consistent with our findings, expression of a dominant negative form of the Wnt receptor Frz8 also resulted in perturbed pancreatic growth and reduced numbers of p48 cells but did not block endocrine differentiation [34]. We did not observe changes in the known Wnt targets *cyclin D1 *or *c-myc*, however we did find that *N-myc*, a potent regulator of proliferation, was significantly down regulated in the β-catenin KO [35-37]. A recent study examining the role of Wnt signaling in lung development demonstrated that like *c-myc*, *N-myc *is a direct transcriptional target of β-catenin/Tcf [10].

Consistent with our observed exocrine deficiency, we observed reduced mRNA levels of numerous exocrine markers in β-catenin mutants. However at E14.5, prior to any visible exocrine hypoplasia, we were surprised to see a striking increase in mRNA levels of several markers of exocrine differentiation including pancreatic elastase3b (*Ela3b*) and amylase 2 (*Amy2*). *Ela3b *was represented 5 times on the chip and was highly up regulated at e14.5 in each. If we relax the stringency of our clustering analysis, numerous other proteinases were also upregulated at E14.5 in 3 out of 4 samples. These include elastase2 (*Ela2*), trypsinogen 16 (*Trygn16*), trypsin 4 (*Try4*), carboxypeptidase A3 and B1 (*Cpa3, Cpb1*), and serine protease 2 (*Prss2*). By E16.5, when there is obvious exocrine hypoplasia, all of these exocrine markers are down regulated, demonstrating that their increased expression at E14.5 is only transient.

Analysis of protein levels by immunohistochemistry show that carboxypeptidase A and amylase protein levels are reduced at E12.5 and E15.5. This could suggest that the observed increase in exocrine markers is only at the mRNA level, or that this is an artifact of microarray analysis. Given the QPCR data demonstrating increases in elastase and amylase transcript at E14.5 and the fact that there is a substantial increase in eight separate exocrine proteinases, it seems unlikely that this is a microarray artifact. There are several possible explanations for a transient increase in exocrine transcripts at E14.5. One possibility is that the loss of a mitogenic Wnt signal triggers a failed attempt at differentiation. Failure to effectively initiate an exocrine differentiation program might be due to reduced levels of p48/PTF1 in the β-catenin mutant, which is known to be critical for the transcriptional activation of many exocrine genes. A similar observation was made in the case of the development of Pdx1 deficient mice, where some limited endocrine (glucagon) and exocrine gene expression was observed even though pancreas development was largely arrested. In addition, although p48/PTF1 is necessary for exocrine pancreas development, it is unlikely that all exocrine genes are direct targets of p48/PTF1. It is also possible that an increase in exocrine markers at E14.5 via microarray analysis is because some remaining β-catenin + cells increase exocrine gene expression in attempts to compensate for the global exocrine loss of differentiation of exocrine progenitor cells. This is consistent with the concept that loss of a mitogenic signal can cause perturbations in proliferation, cell death or differentiation. Our immunostaining does not support this since the remaining β-catenin + cells did not show increased levels of pancreatic enzyme expression. Taken together our data suggests that proliferation and differentiation, but not apoptosis are effected in β-catenin mutants. Moreover, the fact that some exocrine markers are reduced whereas others are elevated suggesting that β-catenin signaling is key at E14.5 to maintain a proper balance between proliferation and differentiation of the exocrine lineage.

In adult mice that survived past 30 days, we observed several instances wherein the pancreatic parenchyma appeared to be partially replaced by tissue that histologically resembles the liver and expresses albumin. This suggests the possibility of hepatic transdifferentiation suggesting that β-catenin function is required to maintain a pancreatic phenotype. Our findings cannot exclude the possibility that disruption of normal pancreatic development caused by loss of β-catenin could have induced nascent hepatocytes to migrate to this area. The phenomenon of hepatic transdifferentiation has been described in other models of severe acinar cell loss including copper or methionine depletion and subsequent repletion but the molecular mechanisms underlying transdifferentation in adults are not well known [38,39]. A hint into a possible mechanism comes from our microarray analyses. We have found that at E16.5, there is a substantial increase in the expression of Hoxb4 in the β-catenin mutant pancreas. This homeotic transcription factor is normally expressed the gut epithelium anterior to the Pdx1 domain and its misexpression in the pancreas suggests that these cells may have undergone some degree of homeotic transformation to an anterior fate [40 and Anne Grapin-Botton, personal communication]. Consistent with this, over expression of Wnt1 using the Pdx1 promoter causes a phenotype suggesting a posterior transformation of the proximal foregut [41]. These data coupled with the observed emergence of albumin expressing cells in the adult pancreas, suggests that β-catenin may be necessary for maintaining positional identity of pancreatic endoderm along the anterior-posterior axis.

The FGF pathway has been shown to regulate exocrine pancreatic development and our finding of down regulation of *Fgfr2 *is concordant with this [42,43]. Components of the hedgehog, and activin signaling pathways that are implicated in endocrine, but not exocrine development, are reduced in β-catenin mutants [44,45]. Specifically, Activin receptor 1c and Hhip1, are reduced in β-catenin mutants, suggesting that these signaling components and pathways may also play a role in exocrine development [46].

## Conclusion

In summary, our studies support a model wherein β-catenin regulates proliferation and differentiation of the developing exocrine pancreas through its role in the Wnt signaling pathway. This observation is consistent with the findings in human exocrine cancers wherein β-catenin/Wnt signaling is inappropriately active and likely supports uncontrolled proliferation. Further studies are required to precisely define the Wnt target genes critical to proliferation of developing acini and to understand the signals that regulate Wnt signaling functions. Our findings also raise the question of whether modulation of β-catenin/Wnt signaling in the adult pancreas could enhance recovery from injury following diseases such as acute pancreatitis, and/or alter the abnormal balance between proliferation and differentiation observed in pancreatic cancer.

## Competing interests

The author(s) declare that they have no competing interests.

## Authors' contributions

JMW carried out pancreata harvest, helped conceive the study, analyze data and draft the manuscript. FE performed the dorsal explant studies and immunohistochemistry, designed figures and assisted with data analysis. GB analyzed all histopathology analyses and assisted with apoptosis assays. BJA performed microarray experiments and bioinformatics. WS performed genotyping, harvested pancreata, prepared samples for microarray analyses and performed quantitative RT-PCR. CC performed genotyping and immunohistochemistry. AS performed immunohistochemistry and apoptosis assays. SDL helped conceive portions of the study, analyze data, and draft the manuscript. AML conceived the study, reviewed and analyzed all data and drafted the manuscript. All authors read and approved the final manuscript.
